# Supplementary material for: Exploring multisite heterogeneity of human basal cell carcinoma proteome and transcriptome
Source: PLoS One. 2023 Nov 10;18(11):e0293744. doi: 10.1371/journal.pone.0293744 (PMC10637653; doi:10.1371/journal.pone.0293744)
Supplement: S1 File — (DOCX) [file pone.0293744.s001.docx]

**Exploring Multisite Heterogeneity of Human Basal Cell Carcinoma**

**Proteome and Transcriptome.**

Ariel Berl, MD^1†*^, Ofir Shir-az, MD^1†^, Ilai Genish, BSc^2†^, Hadas Biran, MSc^3^, Din Mann, MD^1^, Amrita Singh, PhD^4^, Julia Wise, BSc^4^, Vladimir Kravtsov, MD^5^, Debora Kidron, MD^5^, Alexander Golberg, PhD^4*^, Edward Vitkin, PhD^2^, Zohar Yakhini, PhD^2,3*^, Avshalom Shalom, MD^1^

**Supplementary materials**

**S1. Methods**

***Isolating total RNA from tissue using lysis buffer***

The EZ-RNA II kit (Biological Industries, Beit Haemek Ltd., Beit Haemek, Israel) was used to isolate the RNA. The cut tissue was homogenized in the denaturing solution (0.5 ml/50–100 mg tissue) using Bead Beater (BioSpec Products, Inc., Bartlesville, OK) followed by phase separation using 0.09 ml 1–bromo–3–chloropropane (BCP) and 0.4 ml water-saturated phenol, provided in the kit. The RNA was precipitated using isopropanol and later washed with 75% ethanol. The RNA pellets were air dried and dissolved in DEPC-treated water.

***RNA sequencing***

The concentration and integrity of the RNA were determined using Qubit 4 Fluorometer (Thermo Fisher Scientific, Pleasanton, CA) using a 4200Tape Station System (Agilent Technologies, Santa Clara, CA), respectively. cDNA was generated with the KAPA RNA HyperPrep Kit with RiboErase (HMR) (Roche, Basel, Switzerland). Library size was confirmed with High Sensitivity D1000 ScreenTape® on a 4200 TapeStation and the concentration was determined using Qubit 4 Fluorometer. TruSeq RNA single Indexes Set A (12 indexes, 48 samples) was used to construct 24 libraries. Libraries were multiplexed and sequenced on a HiSeq 500 (Illumina, San Diego, CA) to generate 400 million single-end 150-bp reads per library.

***Protein extraction***

The EZ-RNA II kit was used for protein isolation. Each sample of tissue was homogenized in the denaturing solution (0.5 ml/50-100 mg tissue) using Bead Beater (BioSpec Products, Inc., Bartlesville, OK) followed by phase separation using 0.09 ml of BCP and 0.4 ml water-saturated phenol, provided in the kit. The proteins were precipitated using isopropanol and washed later with 0.3 M guanidine HCl in 95% ethanol. The protein pellets were air dried and taken for proteomic analysis.

***Protein sample preparation***

The samples were placed in 8M urea, 400 mM ammonium bicarbonate, 10 mM dithiothreitol (DTT), vortexed, sonicated for 5 minutes at 90% with 10-10 cycles, and centrifuged. The amount of protein was estimated using Bradford readings. 20 µg protein from each sample was reduced at 60ºC for 30 min, modified with 37.5 mM iodoacetamide in 400 mM ammonium bicarbonate (in the dark at room temperature, for 30 min.). The sample was then digested in 2 M urea, 100 mM ammonium bicarbonate with modified trypsin (Promega Corporation, Madison, WI) at a 1:50 enzyme-to-substrate ratio, overnight at 37^o^C. A second digestion with trypsin was done for 4 hours at 37^o^C, under the same conditions.

***Mass spectrometry analysis***

The tryptic peptides were desalted using C18 tips (Harvard Apparatus, Holliston, MA) dried and re-suspended in 0.1% formic acid. The peptides were resolved by reverse-phase chromatography at 0.075 mm, 180 mm fused silica capillaries (J&W Scientific, Folsom, CA) packed with reprosil reversed-phase material (Dr. Maisch GmbH, Ammerbuch-Entringen, Germany). The peptides were eluted with a linear 5%–28% gradient for 180 minutes, 28%– 95% gradient for 15 minutes, with 95% acetonitrile, with 0.1% formic acid in water, at flow rate of 0.15 μl/min. Mass spectrometry was performed with Q-Exactive Plus mass spectrometer (MS) (Thermo Fisher Scientific, Pleasanton, CA) in a positive mode, using a repetitive full MS scan followed by collision-induced dissociation of the 10 most dominant ions selected from the first MS scan. The mass spectrometry data from all the biological repeats were analysed using MaxQuant software 1.5.2.8 vs. the human proteome from the UniProt database with 1% FDR. The data were quantified by label-free analysis using the same software, based on extracted ion currents of peptides, enabling quantitation from each LC/MS/MS run for each peptide identified in any of the experiments.

In addition, one sample, not related to the dataset, was sent through the liquid chromatography-mass spectrometer (LCMS) 5 times and the correlation between each two different outputs was used to measure the LCMS machine "noise".

***Proteomics and transcriptomic data analysis***

Data were analysed based on *python* (ver. 3.7.4) using *pandas* and *scipy* packages. The visualizations were generated using *matplotlib* package.

***RNA-seq alignment***

We used STAR (version 2.7.3a) [30] to process FASTQ files and produce BAM files (for mutation calling) and gene counts (for RNA-seq differential expression analysis). The data set was composed of 9 gene expression profiles of BCC tumors from 3 patients (3 samples from each tumor).

***Correlations Histograms and Distributions***

The Bayesian probability of misclassifying the correlation value from Group 2 as sampled from Group 1 was estimated per each value bin (**Fig. 2, 5**) separately and then totalled, as described in **Eq. 1**:

$$p\left( v assigned to Group1 | v\in Group2 \right)==\sum_{Bi\in\left\{ \begin{aligned} All value bins with \\ non-zero Group2\mathrm{density} \end{aligned} \right\}} \frac{\left[ Group1 density in Bi \right]}{\left[ Group1 density in Bi \right] + \left[ Group2 density in Bi \right]} (Eq.1)$$

***RNA-seq differential expression analysis***

Gene expression data were normalized to counts per million. Genes with an average normalized expression level below 10 per million were filtered out. The heterogeneity threshold was set to log2 (FC)=2. That is, every heterogeneous gene in the list was expressed in one tumor location at least 4-fold more than in some other location of the same tumor. The lists consist of 585, 30, and 191 genes for patients 1, 4, and 7, respectively. For the final list of BCC heterogeneous genes, only genes that appeared in the lists of at least two patients were considered. The final list consisted of 156 genes (p=2.10e-152, computational details and the actual list of genes are listed in the **Supplement** **S3-4**).

***Prediction of RNA mutations***

The significance of the identified positions was assessed with a Jensen-Shannon (JS) distance score. The locations with a high JS score are likely to be somatic SNPs in the tumor (further depicted in the **Supplement S2. RNA analysis**). For instance, samples obtained from patient 7 in chromosome X, genome position 70452749, demonstrated a nucleotide distribution of (0,30,1,0, respectively for A, C, T and G) in the middle region of the tumor, and an almost opposite distribution (0,2,12,0) in the tumor centre, leading to maximum pair-wise JS distance of 0.77.

**S2. RNA analysis**

***Details of computation of Jensen-Shannon (JS) distance score between SNPs***

For every pair of samples, the *mutation calling* algorithm (see next section for pseudocode) uses the Pysam Python package [49,50] to run through all genome locations in the BAM files (produced by STAR, version 2.7.3a) [30]. At every location, it uses the pileup function to load all reads that cover this location and have a minimal base quality (min_base_quality) of 13. Then, it detects locations in which both samples have at least 10 reads with an identified nucleotide (‘A’, ‘C’, ‘G’ or ‘T’, not ‘N’). Among these locations, the algorithm registers the allele distributions that follow one of the following conditions: (i) At least one of the samples has more than one allele. This is defined as true if the most redundant allele frequency is less than 0.99 and there are at least 5 reads with non-major nucleotides. (ii) Each sample has one allele, but it is a different allele for each sample.

Finally, for each registered location, a JS score is computed using the *scipy* package [51]. Specifically, for a vector $p$ listing the distribution of nucleotides in one sample, and a vector $q$ listing the distribution of nucleotides in a second sample from the same tumor, the JS score is computed by applying the formula: $JS=\sqrt{\frac{D(p||m)+D(q||m)}{2}}$, where $m$ is the pointwise mean of $p$ and $q$ and $D$ is the Kullback-Leibler divergence, which is defined as: $D(P||Q)=\sum_{x\in\{A,T,C,G\}} \left[ P\left( x \right)log \frac{P(x)}{Q(x)} \right]$. Then, locations with JS≤0.6 are filtered out. Finally, to receive more meaningful mutations, we narrowed down the received list to include only inside-gene locations.

***Pseudo-code of RNA-seq mutation calling algorithm***

K = minimum number of reads with base quality score > 13 (K=10)

Z = major allele frequency threshold (Z=0.99)

W = minimal number of reads with minor alleles (W=5)

Y = JS score threshold (Y=0.6)

1. For every genome location:
   1. If both samples have at least K reads with quality score > 13 in this location:
      1. Read nucleotides in the location.
      2. Remove unknown nucleotides (keep only ‘A’, ‘C’, ‘G’ and ‘T’, remove ‘N’).
      3. If both samples have at least K reads with ‘A’, ‘C’, ‘G’ or ‘T’ in the location:
      4. If for at least one of the samples, the major allele frequency is <Z and the number of non-major nucleotides is at least W:
2. Register the allele distributions of both samples in this location.
   - 1. Else if there are 2 different major alleles in the samples:
3. Register the allele distributions of both samples in this location.
4. For each registered location, compute the JS distance.
5. Filter out locations with JS < Y.
6. Filter out locations outside of genes.

**S3. Details of computation of p-value for the size of the heterogenous gene set**

To estimate the significance of the obtained set of heterogenous genes, we developed the following computational approach, which was inspired by Wang et al. [52].

Let $N$ be the overall number of genes; let $S_{i}$ be the set of heterogenous genes of patient i, and let $K_{i}$ to be the size of this set: $K_{i}=|S_{i}|$ ($i\epsilon\{1,2,3\}$). Then $S_{ij}$ is defined as the intersection of $S_{i}$ and $S_{j}$, i.e., $S_{ij}=S_{i}\cap S_{j}$ and $K_{ij}$ is the size of this set: $K_{ij}=|S_{ij}|$. Similarly, $S_{ijz}$ is the intersection of $S_{i}$, $S_{j}$ and $S_{z}$ ($S_{ijz}=S_{i}\cap S_{j}\cap S_{z}$), and $K_{ijz}$ is the size of this set: $K_{ijz}=|S_{ijz}|$.

Let $M$ be the set of genes heterogenous for at least 2 patients. Formally: ${M=S}_{12}\cup S_{13}\cup S_{23}$. The null assumption is that all sets are picked randomly and uniformly from the set of genes. Following this assumption, the probability (p-value) of receiving a set of genes of a certain size is the probability that the size of $M$ is as observed or higher:

$$p-value\left( \left| M \right|=m \right)=P(|M|\geq m) = \sum_{m_{0}=m}^{|S_{1}{\cup S}_{2}{\cup S}_{3}|} P(\left| M \right|=m_{0})$$

For each value $m_{0}$:

$$P\left( \left| M \right|=m_{0} \right)= \sum_{w=0}^{\left( K_{1},K_{2} \right)} P(\left| M \right|=m_{0} | K_{12}=w)\cdot P\left( K_{12}=w \right)=\sum_{w=0}^{\left( K_{1},K_{2} \right)} \sum_{z=0}^{min(K_{3},w)} \left[ P(\left| M \right|=m_{0} | K_{12}=w, K_{123}=z)\cdot P(K_{123}=z|K_{12}=w)\cdot P\left( K_{12}=w \right) \right]$$

where:

$$P(\left| M \right|=m_{0} | K_{12}=w, K_{123}=z)={HG}_{(N-w,K_{3}-z,K_{1}+K_{2}-2w)}(m_{0}-w)$$

$$P(K_{123}=z|K_{12}=w)={HG}_{\left( N,w,K_{3} \right)}(z)$$

$$P\left( K_{12}=w \right)={HG}_{\left( N,K_{1},K_{2} \right)}(w)$$

and ${HG}_{\left( N,K,n \right)}(x)$ is the probability mass function of the hypergeometric distribution with the parameters $N,K,n$ for an observed variable $x$.

**S4. List of 156 heterogenous genes in BCC tumors**

ENSG00000167755, ENSG00000165272, ENSG00000124429, ENSG00000167914, ENSG00000164687, ENSG00000167656, ENSG00000197641, ENSG00000088726, ENSG00000159516, ENSG00000158246, ENSG00000092295, ENSG00000186832, ENSG00000137975, ENSG00000166535, ENSG00000182489, ENSG00000145283, ENSG00000162366, ENSG00000163207, ENSG00000162069, ENSG00000169035, ENSG00000163202, ENSG00000171711, ENSG00000126233, ENSG00000206073, ENSG00000165799, ENSG00000143631, ENSG00000188508, ENSG00000178934, ENSG00000235942, ENSG00000204421, ENSG00000100170, ENSG00000143320, ENSG00000124466, ENSG00000129451, ENSG00000143520, ENSG00000084110, ENSG00000163235, ENSG00000197084, ENSG00000119125, ENSG00000198074, ENSG00000211459, ENSG00000203785, ENSG00000133710, ENSG00000074211, ENSG00000170426, ENSG00000133477, ENSG00000158825, ENSG00000143556, ENSG00000121552, ENSG00000241794, ENSG00000187180, ENSG00000143546, ENSG00000186395, ENSG00000105427, ENSG00000136695, ENSG00000189143, ENSG00000075673, ENSG00000139988, ENSG00000263934, ENSG00000196734, ENSG00000268104, ENSG00000148600, ENSG00000129455, ENSG00000102837, ENSG00000119862, ENSG00000124102, ENSG00000147689, ENSG00000111907, ENSG00000175315, ENSG00000163209, ENSG00000134757, ENSG00000144452, ENSG00000140519, ENSG00000166396, ENSG00000197632, ENSG00000159871, ENSG00000159455, ENSG00000111319, ENSG00000136155, ENSG00000118520, ENSG00000118322, ENSG00000167768, ENSG00000163216, ENSG00000167769, ENSG00000174564, ENSG00000108244, ENSG00000210082, ENSG00000281383, ENSG00000167757, ENSG00000124107, ENSG00000135114, ENSG00000196805, ENSG00000006555, ENSG00000215853, ENSG00000188373, ENSG00000105664, ENSG00000163220, ENSG00000171954, ENSG00000186844, ENSG00000136688, ENSG00000085831, ENSG00000124882, ENSG00000139433, ENSG00000167759, ENSG00000203782, ENSG00000197953, ENSG00000118402, ENSG00000159527, ENSG00000170786, ENSG00000185640, ENSG00000167741, ENSG00000179477, ENSG00000169474, ENSG00000205420, ENSG00000104055, ENSG00000183760, ENSG00000119411, ENSG00000169436, ENSG00000188089, ENSG00000244617, ENSG00000183347, ENSG00000203786, ENSG00000189001, ENSG00000175121, ENSG00000170465, ENSG00000184292, ENSG00000165169, ENSG00000169583, ENSG00000179148, ENSG00000188505, ENSG00000170423, ENSG00000175793, ENSG00000185966, ENSG00000185479, ENSG00000069011, ENSG00000127249, ENSG00000105131, ENSG00000088386, ENSG00000178372, ENSG00000057149, ENSG00000172548, ENSG00000172867, ENSG00000168703, ENSG00000136826, ENSG00000166634, ENSG00000171476, ENSG00000134760, ENSG00000161249, ENSG00000109182, ENSG00000169469, ENSG00000244094, ENSG00000286342, ENSG00000113296, ENSG00000121413, ENSG00000088002, ENSG00000105388

**S5. Genome positions with significantly different allele frequencies between intra-tumor samples**

See: https://github.com/GolbergLab/BCC_Heterogeneity
