# Supplementary material for: Exploring multisite heterogeneity of human basal cell carcinoma proteome and transcriptome
Source: PLoS One. 2023 Nov 10;18(11):e0293744. doi: 10.1371/journal.pone.0293744 (PMC10637653; doi:10.1371/journal.pone.0293744)
Supplement: Attachment — Submitted filename: Response.Review Comments .docx [file pone.0293744.s002.docx]

**Response on Review Comments to the Author**

2. Has the statistical analysis been performed appropriately and rigorously?

Reviewer #1: No

5. Review Comments to the Author

Please use the space provided to explain your answers to the questions above. You may also include additional comments for the author, including concerns about dual publication, research ethics, or publication ethics. (Please upload your review as an attachment if it exceeds 20,000 characters)

Reviewer #1: The authors have presented a molecular characterization of basal breast carcinoma and have discussed about spatial heterogeneity of its proteome. While their arguments are reasonable and their claims are somewhat expected, I have some doubts in relation to their statistical experimental design:

1. I cannot see any reason for such a restricted sample number. Even though their samples are apparently well characterized, using 9 samples for proteome analysis and 3 samples (!!) for transcriptomics is extremely restricted, even more if the main discussion is about heterogeneity. There is no attempt to calculate any measure of statistical power in their design. I suggest to consult with an expert statistician to improve on experimental design in order to have data that is actually able to support their assertions.

2. Along these same lines, it is not clear why if the authors already had 9 experimental samples, they used only 3 in the transcriptome analysis that is currently more straightforward to be performed in larger sample counts.

3. I cannot understand the rationale of including SNPs in the characterization of spatial protein heterogeneity.

In brief, the issue under investigation is interesting (though it would be worthy to resort to current experimental techniques in single cell and spatial proteomics), but the poor experimental design and the somewhat disconnected narrative gives the impression (most likely erroneous) that this is just a collection of unrelated experimental results.

We sincerely appreciate the time invested by the esteemed reviewer in reading our article and the thoughtful feedback provided.

We are aware that 27 proteomic samples and 9 transcriptomic samples (3 from each patient) can appear somewhat limited. However, our main null hypothesis was tailored for a small sample set. Specifically, the null hypothesis was that BCC lesions are molecularly homogeneous. Thus, the careful comparative analysis of multiple samples collected from the same lesion even from a very few patients is enough to rebut it in favor of the alternative (that BCC lesions are **not** molecularly homogeneous). Therefore, we respectfully disagree with the reviewer on the statistical quality of the analysis.

Our approach to reject this null hypothesis was by checking for an even stronger alternative. We tested the overlap between the distributions of inter-sample correlations for intra-lesion and inter-patient samples. This overlap, if exists, would indicate cases with the distance between pair of samples from the same lesion as significant as pair of samples from two different patients.

Both for proteomics and for transcriptomics data we show that the distributions of intra-lesion and inter-patient correlations are highly overlapping, clearly rebutting the assumption of lesion homogeneity.

To better emphasis this, we refined (changes are marked in **red** here) the manuscript as following:

“…the meaningful intersection between the histograms implies significant heterogeneity of specific tumors, clearly rebutting the hypothesis of lesion proteomic homogeneity. In other words, in notably many cases, the difference between two proteomic samples extracted from the same tumor of a single patient was as significant as the difference between two proteomic samples extracted from two different patients, sometimes even from two different BCC subtypes. Quantitively, **the Bayesian probability of misclassifying the correlation** (Group 1 vs. Group 2) **was 40.7% and 48.1%** for Spearman and Pearson tests, respectively.”

for proteomics data and

“…the meaningful intersection between the histograms implies significant heterogeneity of specific tumors, clearly rebutting the hypothesis of lesion transcriptomic homogeneity. In other words, in some cases the difference between two RNA samples extracted from the same tumor of a single patient was as significant as the difference between two RNA samples extracted from two different patients with different BCC subtypes. Quantitively, **the Bayesian probability of misclassifying the correlation** (Group 1 vs. Group 2) **was 22.2% and 11.1%** for Spearman and Pearson tests, respectively.”

for transcriptomics data.

Addressing the #2 comment of the reviewer, the decision to perform transcriptomics for 9 out of 27 samples was received both due to project budget considerations and in view of results from the proteomic analysis, that clearly indicated the molecular heterogeneity.

We also want to explain the rationale of including SNPs to respond the comment #3 of the esteemed reviewer. The goal of this analysis was to address another aspect of molecular heterogeneity by checking the possibility to identify newly appeared somatic mutations inside a lesion. If the inherent nature of the lesion is homogeneous, we would not be able to find such mutations. Since this is strictly an intra-lesion analysis, addressing multiple samples collected from the same lesion is sufficient, akin to the approach reported in many single cell studies. Thus, the results are reported separately for each of 3 patients with available transcriptomic data:

“Somatic SNPs were studied by comparing the sequencing data of intra-patient locations under the assumption that the distribution of expressed alleles should be similar across skin cells of the same individual [35]. Following this approach, we found 22, 30, and 6 genome locations with JS distance above 0.6 for patients 1, 4 and 7 respectively (Supplement S2).”

We would like to express our sincere appreciation to the esteemed reviewer for their time and effort.

We would also like to thank the esteemed editor for their efforts in reading the article and finding the reviewers. We have carefully studied the feedback and suggestions provided and refined the manuscript accordingly.

We believe that the submitted manuscript provides a valuable contribution to the field. We are open to any further comments from the editor and remain dedicated to ensuring the quality and relevance of our work. Thank you for your ongoing support and guidance throughout this process.
