# Supplementary material for: Exploring multisite heterogeneity of human basal cell carcinoma proteome and transcriptome
Source: PLoS One. 2023 Nov 10;18(11):e0293744. doi: 10.1371/journal.pone.0293744 (PMC10637653; doi:10.1371/journal.pone.0293744)
Supplement: Attachment — Submitted filename: reviewer comments.AG.docx [file pone.0293744.s003.docx]

13 September 2023

Dear Editor,

We would like to express our sincere appreciation to the esteemed reviewer for their time and effort.

We would also like to thank the esteemed editor for their efforts in reading the article and finding the reviewers. We have carefully studied the feedback and suggestions provided and refined the manuscript accordingly.

We believe that the submitted manuscript provides a valuable contribution to the field. We are open to any further comments from the editor and remain dedicated to ensuring the quality and relevance of our work. Thank you for your ongoing support and guidance throughout this process.

Sincerely,

Alexander Golberg

Point by point response.

Reviewer #2: The authors describe a multisite characterization of transcriptomic and proteomic profiles in a limited number of basal cell carcinoma tissues isolated from human patients. They calculated hierarchical clustering and defined two relevant biological groups based on their omic similarities. They detected inter- and intra-tumor heterogeneity. In my opinion, this study is a proof of concept of a particular analytical strategy, but a biological assessment cannot be performed in its current form due to the limited number of data and, consequently, the basic analysis that can be performed on this type of data. I believe it is not suitable for publication in its present form.

We would like to comment on this summary of the esteemed reviewer. In our research we did not perform any hierarchical clustering and neither did we search for biological groups based on sample omics similarities.

The patients were grouped by the pathologist’s diagnosis according to the detected BCC subtype. The samples were grouped according to their patient origin, thus defining intra-tumor group of samples. Our primary goal was to investigate the level of heterogeneity of molecular measurements inside each tumor. The inter-tumor heterogeneity was investigated as a reference group for the observed results.

The main research hypothesis was tailored for a small sample set, specifically, that BCC lesions are molecularly homogeneous. Thus, the careful comparative analysis of multiple samples collected from the same lesion even from a very few patients is enough to rebut it in favor of the alternative (that BCC lesions are not molecularly homogeneous). Such a result has implications on decision made from molecular data obtained from a single-site biopsy.

Mayor concerns
The authors state there is not a widely used strategy to assess heterogeneity and this paper proposes several approaches to do so. I disagree with the authors, since there are several robust techniques that have been applied to BBC such as single cells, spatial proteomics and dedicated computational methods mainly based on artificial intelligence such as machine learning approaches as well as graph neural networks and mutidmensional integration of omics data. Some elegant examples are: Unravelling the landscape of skin cancer through single-cell transcriptomics (10.1016/j.tranon.2022.101557), Single-cell analysis of human basal cell carcinoma reveals novel regulators of tumor growth and the tumor microenvironment (10.1126/sciadv.abm79) and Integrated multi-omics reveals cellular and molecular interactions governing the invasive niche of basal cell carcinoma (10.1038/s41467-022-32670-w). As I have already mentioned, I believe this is a homemade approach to describe a complex phenomenon.

We are grateful for the reviewer for bringing these novel studies to our attention. We added these important recent references and updated the text. Our approach also deals, as mentioned by the reviewers with several sites in the same lesion, which is, indeed, different from the single cells works published, which looks at many cells in the same site. The number of different patients in our work and published single cells studies is similar suggesting similar needed statistical tools to describe inter-patients heterogeneity.

The title needs to be changed: in formal terms, spatial proteomics aims to characterize protein locations and their dynamics at the subcellular level. The evaluation presented in this study analyzed multisite gene expression profiles (3 different samples extracted from a single lesion), not formally a spatial characterization. In addition, the title only mentions proteomic analysis, but in some tumors the transcriptomic lanscape is also presented. Please modify the title to better describe what was performed.

Amended as requested

In my opinion, at least for protein analysis, it would be better from a computational and interpretive strategy point of view to calculate the k-mean in addition to hierarchical clustering.

Again, no clustering was performed in this research. Patients and their samples were grouped according to histopathological diagnosis.

Show the adjusted p-values for the correlation matrix (Spearman and Pearson) and not just the R-squared.

We do not report the coefficient of determination (R-squared) in our analysis. We report correlation coefficients (Spearman-R and Pearson-R).

Since the vector lengths are equal for all analysis pairs, the correlation coefficients are directly correlated with the p-values (i.e. higher the correlation coefficient – lower the corresponding p-value). Therefore, for visualization simplicity we decided to report correlation coefficients only.

The authors mentioned that they detected differences between: "two RNA samples extracted from the same tumor from the same patient" and " between two RNA samples extracted from two different patients with different BCC subtypes". In either of these cases, tumor purity is an important feature that could affect these results, mainly because the authors described that the tumor tissue collected included both tumor tissue and healthy margins. Please note this important histopathologic feature in the analysis.

We agree with the reviewer and added the sentence on the limitation of the sampling and analysing the bulk tissue in the discussion.

In the RNA-seq analysis I guess the authors did not remove those genes that are not expressed in x% of the samples before normalization, that is the reason why they had a large number of RNAs. Please remove uninformative genes from your analysis before normalize raw count data.

We agree that removing lowly-expressed genes is a common and useful practice. However, since we wish to evaluate variability between different samples of a tumor, excluding genes that are only expressed in one sample would be counterproductive. We decided to remove genes with less than 0.5 CPM (counts per million) in all samples (this threshold is advised by [1] and [2]). This did not affect the differential analysis, since we only considered genes that were found in at least two patients, however intersection analysis slightly changed and was updated.

[1] Chen, Yunshun, Aaron TL Lun, and Gordon K. Smyth. "From reads to genes to pathways: differential expression analysis of RNA-Seq experiments using Rsubread and the edgeR quasi-likelihood pipeline." *F1000Research* 5 (2016).

[2] Galaxy training! “2: RNA-seq counts to genes”. URL: [https://training.galaxyproject.org/training-material/topics/transcriptomics/tutorials/rna-seq-counts-to-genes/tutorial.html#filtering-to-remove-lowly-expressed-genes](https://training.galaxyproject.org/training-material/topics/transcriptomics/tutorials/rna-seq-counts-to-genes/tutorial.html%23filtering-to-remove-lowly-expressed-genes)

I believe that this study cannot describe a biological landscape for the limited number of samples evaluated. In other approaches such as single cell, the evaluation of few patients could provide relevant information because thousands of single cells are evaluated, in this case there is only a bulk evaluation and therefore I believe there is not enough data to make biological interpretations.

The goal of this study was to show heterogeneity of the tumor in multiple sites with implications for diagnostics done with single site biopsy. We clarified this in the revised version. The biological interpretation shown is the inter- and intra patient heterogeneity.

I do not believe that "SNP" analysis provides any relevant information. First, I think the correct term is mutational analysis and therefore the authors identified mutations in coding regions, not SNPs. Secondly, I believe that although mutational data are useful for defining subclonal architecture, in this case there is no specific analysis to assess it, and thus any relevant data for the biological questions.

We removed the use of “SNPs” and replaced it with “somatic SNVs” and “point mutations”. As to the second remark, we believe that this analysis helps in establishing the heterogeneity of BCC tumors. If the tumors are homogenous, the distribution of different nucleotides in a genomic location should be roughly the same between different samples of the same tumor. For example, let’s say that in one sample we see 2 different nucleotides in the same genomic location: 30 RNA strands with “A” and 70 RNA strands with “C”. These could be the two germline alleles of the individual. However, if this is the case, we would expect that in all three samples that belong to the same individual, the distribution of alleles would be roughly the same: 3:7 between “A”s and “C”s. However, if we see a significantly different distribution of “A”s and “C”s (or “G”s or “U”s) in the other samples, then a possible explanation would be that a mutation exists, and is more prevalent in one section of the tumor then in other sections. There are also other possible explanations, for example that there’s a copy number variation that affects this distribution or that another biological process affects the expression of germline alleles. However, all of these possible explanations rely on the fact that different samples of the same tumor behave differently (whether due to a point mutation or for another reason), so they all contribute to the heterogeneity explanation.

Show the dendograms of the hierarchical clustering analysis.

No clustering was performed in this research.

Indicate in the PCA analysis the % of variance explained by each component.

Amended as requested.

Improve figure quality

Amended as requested.
